# Establishment of the Society for the Advancement of Neuroscience and Psychiatry in Residency Research Education (Synapse): An Organization to Promote Research Training in Residency

**DOI:** 10.1007/s40596-026-02312-0

**Published:** 2026-03-04

**Authors:** Heather Ward, Olusola Ajilore, Youngsun Cho, Jessica Cooper, Boadie Dunlop, Andrew Miller, Noah Philip, Brandon Pruett, Stephan Taylor, Audrey Tyrka, Alecia Vogel, David Goldsmith

**Affiliations:** 1 Vanderbilt University Medical Center, Nashville, TN USA; 2 University of Illinois at Chicago, Chicago, IL USA; 3 Yale University, New Haven, CT USA; 4 Emory University, Atlanta, GA USA; 5 Augusta University, Augusta, GA USA; 6 Brown University, Providence, RI USA; 7 University of Alabama at Birmingham, Birmingham, AL USA; 8 University of Michigan–Ann Arbor, Ann Arbor, MI USA; 9 Washington University in St. Louis, St Louis, MO USA

The physician-scientist workforce has declined for four decades, and in 2020, only 1.3% of psychiatrists identified research as their primary activity, down from 1.6% in 2011 [1]. From 2013 to 2022, the number of National Institutes of Health (NIH) career development awards to physicians was stagnant [2]. Barriers to a physician-scientist career are mounting, as the age at first R01 increases and the future of NIH funding is unclear. The obstacles to physician-scientist training are well established, and there have been many attempts to bolster research training in residency to grow the physician-scientist population [3]. In 2001, the National Institute of Mental Health (NIMH) commissioned a report from the Institute of Medicine (IOM) on obstacles and solutions to physician-scientist training in residency [4]. One obstacle was fragmented research training throughout residency. As a result, residency research tracks were developed to provide formalized research training in residency [5–7].

Psychiatry residency research tracks provide substantial protected research time, mentorship, and funding during the earliest stages of building a research career [8]. Many research tracks are funded through the NIH R25 mechanism, which was established over 20 years ago to support educational initiatives intended to enhance the pipeline of well-trained researchers and to diversify the biomedical workforce. The NIMH began funding R25s to ensure continued engagement in research through residency research tracks. Rather than stepping away from research during residency training, the R25 provided protected time so residents could stay engaged in research, improving retention of physician scientists and reducing the time to a K award and first R01.

Despite the R25 programs, the number of psychiatrists engaging in research continues to decline. Of the over 350 psychiatry residency programs in the USA, there are currently 17 NIMH-funded R25 research tracks. There are also many non-R25-funded research tracks, which must find alternative ways to support research, including leveraging departmental support and/or funding from graduate medical education. Thus, for many programs, the hurdles to support residents engaging in research remain high.

Even with dedicated resources, residency can be a precarious time for physician-scientists as they balance demanding clinical and research obligations (Fig. 1). Student loan repayment, family, and financial considerations must be weighed [9]. Although research tracks support residents, there is significant heterogeneity among tracks in their structure, resources, and outcomes.

**Fig. 1:**
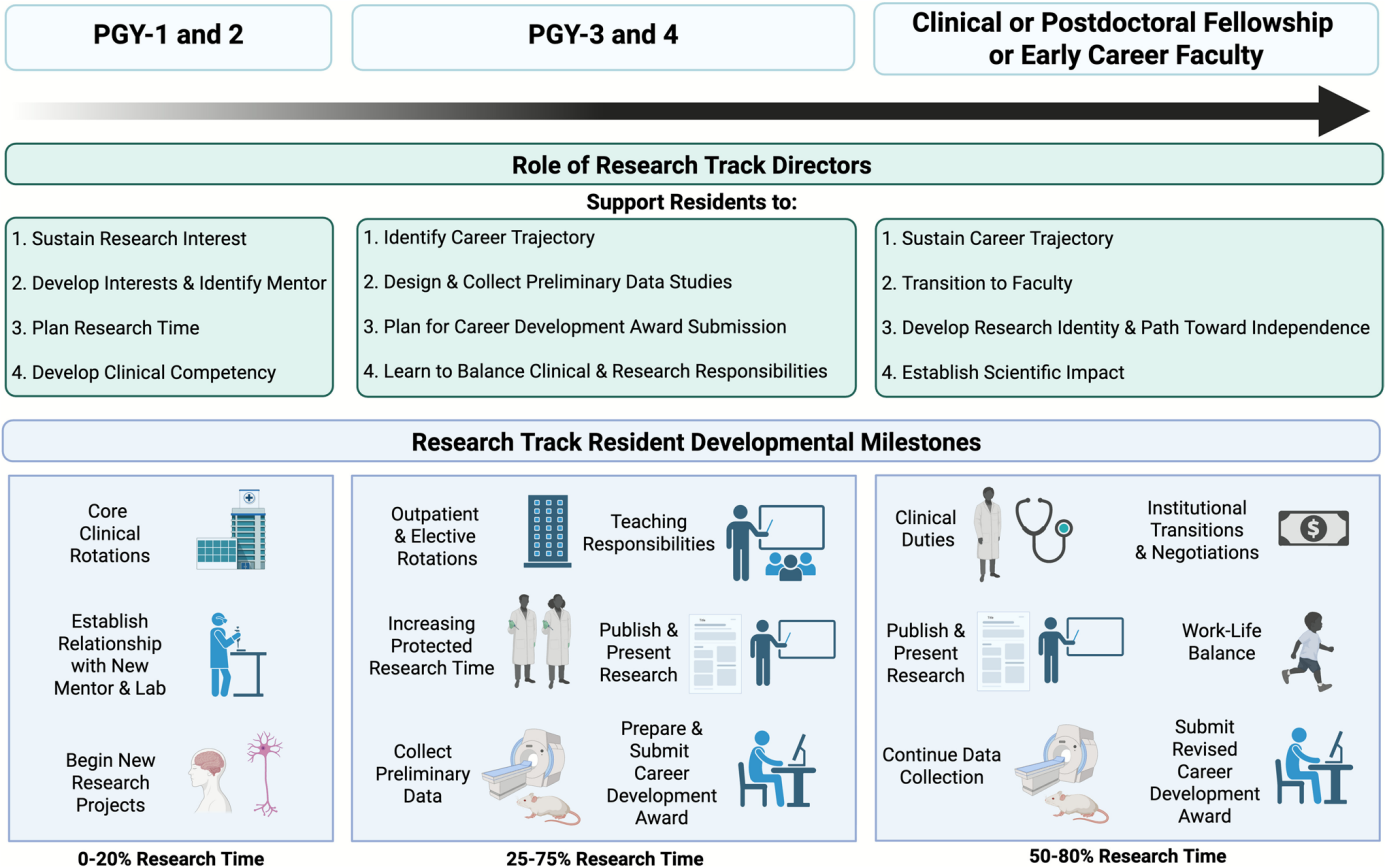
Role of the Society for the Advancement of Neuroscience and Psychiatry in Residency Research Education. Even in a research track, the residency years for physician-scientists are precarious, as they must balance demanding clinical training with research productivity, funding acquisition, and career transitions, shown with a range of protected time provided in research tracks. Research track directors support residents throughout training and into faculty positions as they navigate these obstacles. PGY, post-graduate year. Created with BioRender.com

Categorical psychiatry residency programs must follow guidelines from the Accreditation Council for Graduate Medical Education (ACGME) [10], which governs clinical training requirements for residency programs, and the American Board of Psychiatry and Neurology, which determines eligibility for board certification. Although psychiatry research tracks are embedded within categorical programs and must abide by ACGME and ABPN requirements, there are no established guidelines for research training for aspiring physician-scientists. While physician-scientists must first be fully trained clinicians, the balance of clinical and research time and what clinical standards should be applied to research track residents is not currently considered, despite recommendations from the IOM report [4].

The American Association of Directors of Psychiatric Residency Training (AADPRT) organizes psychiatry training directors to facilitate sharing knowledge, education, and best practices among residency programs. However, there is no organization of research track directors with similar goals of shared resources and best practices. Research track directors are generally physician-scientists whose primary identities are in research and who may not otherwise be involved in AADPRT.

To fill this gap, we established the Society for the Advancement of Neuroscience and Psychiatry in Residency Research Education (Synapse), an organization of psychiatry residency research track directors. The mission of Synapse is “To build a network of research track directors to promote and share best practices in psychiatry residency research training and to advocate for the needs of aspiring physician-scientists in residency training.” Our inaugural meeting was held at the Society of Biological Psychiatry (SOBP) annual meeting in Toronto, Canada, in April 2025 and was attended by representatives from academic psychiatry departments included in the authorship of this paper. Synapse is an organization independent from SOBP. Here we outline the primary objectives of Synapse and key areas for future collaboration.

At our initial meeting, there was consensus regarding the urgency to address the shortage of physician-scientists in psychiatry to advance treatment for psychiatric disorders. To achieve this goal, program directors were enthusiastic about sharing best practices and creating synergy between programs. Discussion focused on four key areas: (1) track structure; (2) resources; (3) predictors of success; and (4) post-residency transitions.

## Track Structure

There is considerable heterogeneity between programs in how protected research time is allocated. Some programs have separate match processes within the Electronic Residency Application Service, while others match from categorical programs. Other programs allow for child and adolescent fellowship training interspersed with dedicated research time. In all cases, collaboration with categorical program directors is critical to the track’s success.

Most research tracks provide 12–15 months of protected research time concentrated in the final 2 years of residency [11]. The first 2 years of training are dominated by intensive clinical requirements, yielding little flexibility for research rotations. Research track directors agreed that protected research time during the first 2 years was critical to residents’ sustained research productivity, identification as a physician-scientist, and retention in the research track, without compromising comradery with categorical co-residents. Although programs tend to have substantial protected time in post-graduate years (PGY) 3 and 4, there is variability in the time allocated each year, and some programs allow for flexibility to match the specific individual needs.

To meet the same clinical requirements as categorical residents, some programs have rotations that meet requirements *and* provide critical research training. For example, rotations on an inpatient clinical research unit count toward required inpatient rotation months. Depending on the type of research (e.g., evaluating clinical trial participants), research time may also count toward required clinical training.

Overall, the significant variability between tracks can create confusion for applicants. Structural differences pose challenges in quantifying the number of research track positions and measuring outcomes nationwide. Despite having research tracks for many years, there is a dearth of data for how many residents receive additional research training, demographics of these residents, and research backgrounds (e.g., numbers of MD/PhDs and publication history), a key challenge also identified in the 2001 IOM report [4]. Synapse aspires to systematically collect these data, in accordance with IOM recommendations, to better characterize who is seeking research training, who is underrepresented in research training, and predictors of research track success. These will provide key data to advocate for the needs of research track residents moving forward.

## Resources

There is also substantial heterogeneity in resources provided by research tracks, which may be related to funding sources. Research tracks support mentorship, protected research time, research expenses (e.g., equipment, conference travel, coursework), and administrative time. The NIH R25 is the most traditional funding mechanism for this, though there are far fewer R25 tracks than residency programs. Funding via other mechanisms (e.g., ACGME funding, departmental funding sources, NIH T32s in the PGY-4 or postdoctoral years) contributes to variability in available resources.

Research funding provided by tracks varies widely and is not always transparent. Most programs provide research resources in the form of expertise, space and equipment, research supplies, and other institution- and mentor-provided resources. Several tracks provide significant financial support for trainee projects (up to $50,000) over a 4-year residency. Other programs offer research funding on a case-by-case basis, similar to negotiating a start-up package. As an alternative funding model, some research tracks provide an annual salary supplement and rely on mentors to support research expenses. In our meeting, research track directors expressed concern over equity in this situation and how unionization among residency programs may affect research track resources.

Other sources of financial support, such as the NIH Loan Repayment Program, increase a research track resident’s likelihood of receiving a career development award, subsequent R-level awards, and overall retention in a research-focused career [1, 12]. Whether additional support for research track residents increases their success has not been studied, another area of exploration for Synapse. Salary support and research expenses can also serve as a recruitment tool for future faculty. As residents establish a relationship with a mentor, develop a research vision, and collect preliminary data, joining the faculty at that institution provides an appealing opportunity. This allows the resident to maintain momentum, capitalize on existing relationships and institutional knowledge, and avoid uprooting family. Similarly, rather than focusing on external recruitment, which can be time intensive, costly, and low yield, departments might see greater returns by investing internally in their research track residents. The merits of financial support for research track residents are complex, often future oriented, and another area in need of further study to develop best practices, ensure equity, and determine whether they lead to retention in research careers.

Synapse can also play a role in broadcasting resident opportunities for funding or career development, such as the American Psychiatric Association Research Colloquium for Junior Psychiatrist Investigators and NIMH Outstanding Resident Award Program. Synapse can advocate on behalf of trainees to scientific organizations (e.g., SOBP), as well as governing bodies (e.g., AADPRT). Synapse can support both research track residents and even applicants in identifying co-mentors at other institutions for electives, collaborations, and future career development award applications.

## Predictors of Success

A research career may take many forms, including academic NIH-funded investigators or researchers in industry. Similarly, there may be many markers of “success” for research tracks, ranging from graduating from a research track (regardless of whether it leads to a research career) to submitting and receiving a career development award and to maintaining a research career as faculty. In our meeting, research track directors lamented the challenge of predicting success. There was consensus that residents with the highest probability of success were individuals with a clear research vision, relevant research skills, and an engaged mentor to help the resident achieve that vision. Track directors shared examples of residents who were ultimately unsuccessful because of a dramatic change in focus, requiring them to learn new skillsets, which may be particularly challenging in residency. Developing practices around identifying these individuals early and supporting their success was seen as a key unmet need.

Similarly, track directors identified challenges based on a resident’s research background, namely those with MDs compared to MD/PhDs. While MDs generally do not have the depth of research training as MD/PhDs, their focus is often more amenable to clinical translation and may be easier to integrate with clinical care. In contrast, MD/PhDs often have depth in a particular area, which can be a tremendous asset if they aim to continue in that area. Additionally, MD/PhDs are usually older than MDs, bringing up potential challenges related to burnout and financial concerns.

Though there are numerous anecdotal threads to suggest which residents may progress to a career in research, there is increasing interest in harmonizing data and determining metrics of success to empirically evaluate who is most likely to achieve the goals of research track training and how to best support those individuals at risk of abandoning a research career [13].

Regardless of background, track directors also emphasized the importance of setting expectations for trainees and for mentors, consistent with the literature [14]. Trainees should have clear goals and expectations for each year of training. Mentors, especially those without clinical experience, need education in the clinical demands of residency, especially in PGY 1 and 2. A clear understanding of the limited protected research time afforded to research track residents (and how it is substantially less time than a graduate student or post-doc) is key to ensuring a successful experience.

## Post-Residency Transitions

An early milestone of success in launching a research career is receipt of a career development award, most often from the NIH or Veterans Affairs. To achieve this goal, many research tracks encourage and support their residents in submitting a career development award by the end of training or in the years immediately following training. This process is an investment years in the making that requires the establishment of a mentoring relationship, development of training and research plans, collection and analysis of preliminary data, and preparation of the submission. Yet, obtaining a career development award rarely occurs during residency, so young investigators often need bridge funding to support protected research time while submitting or revising their application or awaiting the award. This is even more important for residents who may not be ready to apply for a K award by the end of residency. Institutions have a variety of different mechanisms to provide funding continuity, which may take the form of internal awards/fellowships (e.g., NIH KL2, philanthropic, or foundation fellowships) or T32 post-doctoral training awards that can be combined with an instructor position and clinical work. Further complicating post-residency transitions is how to support residents who want to pursue clinical fellowship training, where there is little protected time for research. Developing best practices for all these scenarios is a key aim of Synapse.

## Conclusion

Psychiatry residency research tracks offer valuable research training and promote physician-scientists. By creating a network of research track directors, we hope to facilitate communication and collaboration to support research trainees across all institutions. There was consensus that Synapse should meet annually or semiannually in-person at the SOBP and American College of Neuropsychopharmacology annual meetings. Synapse can also create a community of physician-scientists in residency who can also meet at national meetings. Directors also agreed to create a listserv and website that collates research track information. More engagement with AADPRT would likely be beneficial for greater collaboration between research tracks and the categorical programs that house them. Finally, it may be appropriate to discuss more flexible clinical requirements for research track residents.

## Data Availability

No datasets were generated or analysed during the current study.
